# An MRI sequence independent convolutional neural network for synthetic head CT generation in proton therapy

**DOI:** 10.1016/j.zemedi.2021.10.003

**Published:** 2021-12-15

**Authors:** Lukas Zimmermann, Barbara Knäusl, Markus Stock, Carola Lütgendorf-Caucig, Dietmar Georg, Peter Kuess

**Affiliations:** 1 Medical University of Vienna, Department of Radiation Oncology, Vienna, Austria; 2 MedAustron Ion Therapy Center, Wiener Neustadt, Austria; 3 Faculty of Engineering, University of Applied Sciences Wiener Neustadt, Austria; 4 Competence Center for Preclinical Imaging and Biomedical Engineering, University of Applied Sciences Wiener Neustadt, Austria

**Keywords:** MRI-only, Proton therapy, Synthetic CT, Transfer learning

## Abstract

A magnetic resonance imaging (MRI) sequence independent deep learning technique was developed and validated to generate synthetic computed tomography (sCT) scans for MR guided proton therapy. 47 meningioma patients previously undergoing proton therapy based on pencil beam scanning were divided into training (33), validation (6), and test (8) cohorts. *T*_1_, *T*_2_, and contrast enhanced *T*_1_ (T1CM) MRI sequences were used in combination with the planning CT (pCT) data to train a 3D U-Net architecture with ResNet-Blocks. A hyperparameter search was performed including two loss functions, two group sizes of normalisation, and depth of the network. Training outcome was compared between models trained for each individual MRI sequence and for all sequences combined. The performance was evaluated based on a metric and dosimetric analysis as well as spot difference maps. Furthermore, the influence of immobilisation masks that are not visible on MRIs was investigated. Based on the hyperparameter search, the final model was trained with fixed features per group for the group normalisation, six down-convolution steps, an input size of 128 × 192 × 192, and feature loss. For the test dataset for body/bone the mean absolute error (MAE) values were on average 79.8/216.3 Houndsfield unit (HU) when trained using T1 images, 71.1/186.1 HU for T2, and 82.9/236.4 HU for T1CM. The structural similarity metric (SSIM) ranged from 0.95 to 0.98 for all sequences. The investigated dose parameters of the target structures agreed within 1% between original proton treatment plans and plans recalculated on sCTs. The spot difference maps had peaks at ±0.2 cm and for 98% of all spots the difference was less than 1 cm. A novel MRI sequence independent sCT generator was developed, which suggests that the training phase of neural networks can be disengaged from specific MRI acquisition protocols. In contrast to previous studies, the patient cohort consisted exclusively of actual proton therapy patients (*i.e.* “real-world data”).

## 1 Introduction and purpose

Due to its superior soft tissue contrast, MRI is an essential image modality in radiation oncology for organ at risk (OAR) and target delineation. This applies to both photon and particle therapy treatment routines. Regardless, computed tomography (CT) information is still required for treatment planning as the electron density (photon) or stopping power (protons) information is the basis for dose calculation. This implies that CT-MR image registration is mandatory, which introduces a geometric uncertainty that is propagated throughout the whole treatment planning process. Therefore, the concept of an MRI-only planning approach has been intensively investigated for the past decade. For photon therapy, different treatment sites were successfully investigated with promising candidates for wider clinical implementation [1], [2], [3], [4], [5], [6], [7], [8], [9]. Likewise, for proton therapy, several studies are available [6], [10], [11], [12], [13].

Recent research in MRI-only simulation and MRI guided radiation therapy has resulted in commercial solutions from vendors. This covers dedicated MR scanners with adequate positioning devices and fixation tools [14]. Furthermore, MR-linear accelerators (MR-linac) for tumour motion tracking during treatment are gaining prominence [15], [16]. Besides the necessary hardware requirements, software for accurate distortion correction and sCT generation is needed to accurately define patient geometry and HU. In addition to the MR-only workflow approach based on commercial solutions, the use of clinical MR images acquired during treatment for dose recalculation and subsequent uncertainty estimation shows major potential in optimising particle beam therapy. It should be noted that, when using an sCT instead of a pCT, the potential differences have a more severe impact for particle treatments than photon treatments. This is primarily due to the different depth dose characteristic energy deposition, but also due to the sensitivity to density changes along the beam path. Furthermore, literature on proton therapy lacks information on the impact of immobilisation masks, which are not visible on MRIs.

Recent research on sCT generation has moved from atlas-based techniques to the application of deep learning algorithms. Several methods have been investigated, ranging from fully convolutional neural networks (CNNs) [3], [4], [6] to generative adversarial networks (GANs), including the popular CycleGAN method [7], [11]. CycleGAN has the advantage of learning mapping of unaligned images; however, compared to a traditional supervised learning schema, it is not directly visible in the metric results.

Most studies are based on datasets and training methods using a single scanner and sequence for training and subsequent validation. This disregards the potential of neural networks to extract complex features describing the general shapes from differently contrasted images. A major drawback of such techniques is the lack of applicability of trained models with other MRI sequences or even other scanners (*i.e.* model generalisation for neural network based techniques).

In a recent study, model generalisation was achieved for *T*_2_-weighted sequences by using data that was acquired with different field strengths and from vendors that were not included in the training set [17]. This raises the question if deep learning models can adapt even better to multiple inputs if different MRI sequences are utilised during the training phase. As image protocols, and with that MRI sequences, are adapted with time, it seems inevitable that the output quality of sCTs generators should be unaffected by such changes.

This study investigates the possibility of using multiple MRI sequences to train a deep learning model. Such a technique has the potential to overcome the limitation of sequence dependent sCT quality, paving the way towards a broad clinical implementation in proton therapy. Besides a metric evaluation of the generated sCTs, a dosimetric comparison was performed using clinically applied proton plans. In addition, spot difference maps were calculated.

The application of a universally trained model is beneficial for institutions that acquire multiple MRI sequences but may omit certain sequences due to the patients’ condition or history. Furthermore, a change of the clinically applied imaging protocol (*e.g.* only performing T1CM, except the patient does not tolerate contrast media) would no longer require the training of the neural network with a complete new dataset. This is especially important in upcoming treatment centres which are still improving their clinical workflow. To our knowledge, this rationale was not the driving force in previous literature, but it is of particular importance for the clinical application of sCTs.

## 2 Materials and methods

### 2.1 Patient cohort and image acquisition

In total 47 meningioma patients treated with proton therapy were included in this study. All patients received MR (Ingenia 3.0, Philips, Netherlands) and CT (Big Bore CT, Philips, Netherlands) scans on the same day in treatment position using immobilisation devices, like thermoplastic face masks and necessary positioning tools administered during proton irradiation. In more detail the model was trained with data from 33 patients, six patients were used for validation, and eight for testing the model.

All patients were included in a prospective clinical study at the MedAustron Center for Ion Beam Therapy and Research [18], approved by the responsible ethics committee (Niederösterreische Ethikkommission, Austria) under the number GS1-EK-4/350-2015.

The CT sequence was acquired with a peak voltage of 120 kV. The volume was reconstructed with a transversal plane resolution of 0.684 × 0.684 mm^2^ and a slice thickness of 2 mm. For the CT protocol the HU calibration was acquired and validated at the MedAustron Center for Ion Therapy and Research during the commissioning phase using the CIRS electron density phantom (CIRS, Norfolk, US). MRI included *T*_2_- and *T*_1_-weighted sequences, where *T*_1_-weighted was performed with and without contrast medium (CM). Image resolution and slice thickness varied for different sequences between 0.2 × 0.2 and 0.47 × 0.47 mm^2^ and 0.9 and 1.9 mm. Further settings of the sequences are listed in Table 1.

**Table 1:** Used MR sequence parameters.

	*T*_1_	*T*_2_	*T*_1_CM
MR contrast	T1w	T2w	T1w
Sequence type	Spoiled gradient echo	Spin echo	Spoiled gradient echo
TE [ms]	3.0–3.4	237–313	3.4
TR [ms]	5.2–6.7	2500	7.2
Flip angle [°]	12	90	12
Averages	1–3	1–2	2
Orientation	Transversal	Transversal	Transversal
Contrast agent	No	No	Yes

CT images were rigidly registered to all MRIs separately after re-sampling the MRIs to the same voxel dimensions of the pCT. The Elastix implementation in the MICE Toolkit (2020 NONPI Medical AB, Umeå, Sweden) was utilised for the registration.

In the training dataset for all 33 patients, the *T*_1_CM-weighted MRI sequence was included, for 24 patients the *T*_1_-weighted sequence, and for 32 patients the *T*_2_-weighted sequence. For the validation dataset, all six patients received all three sequences. The eight patients representing the test dataset included 16 treatment plans in total. For one of these patients, the *T*_1_-weighted sequences, and for three other, the *T*_2_-weighted sequence did not fully cover the target structure, subsequently these images were excluded. One additional patient with a Sphenoid wing meningioma was also evaluated to investigate model's limits but was not included in the overall analysis.

### 2.2 Clinical treatment plan creation and prescription

All treatment plans included in this study were clinically approved and delivered, and included three to four beam directions, while the entrance angle differed by at least 20°. Those plans were recalculated on the sCTs. The relative radiobiological effectiveness (RBE)-weighted dose per fraction varied between 1.8 and 2.2 Gy (RBE of protons was assumed with 1.1). Seven of the eight patients received a simultaneous integrated boost (SIB) for initial and boost target volume in 27–30 fractions, while one patient received subsequent irradiation of the two target volumes.

The treatment planning system RayStation v8b (RaySearch, Sweden) employing the Monte Carlo (MC) dose calculation algorithm v4.1 with 10,000 particles per spot and 0.5% statistical uncertainty was employed using a dose grid of 2 mm. Spot spacing was approximately 5 mm while the energy layer spacing was below 3 meV, depending on the proton energy and the use of a range shifter.

All treatment plans included in the dosimetric evaluation study were optimised following the single-field uniform dose (SFUD) approach, assuring a constant dose to the target from the different beam directions. The brainstem and the optical system were prioritised over the target coverage during the treatment plan optimisation process. To all other OARs, for example, the whole brain, the temporal lobes, or the hippocampi, the dose was reduced as much as reasonably achievable while the values for clinical constraints were patient – and anatomy – dependent.

### 2.3 Deep learning model and hyperparameter search

A 3D U-Net architecture [19] with ResNet-Blocks [20] between down- and up-sampling convolutions was used as a baseline for this study, as this provided good results for image-to-image translation tasks [21]. A 4 × 4 convolution with a stride of 2 was applied at the beginning with a base filter set of 64 features. ResNet-blocks employed a bottleneck where the features were first reduced to half of the initial size with a 1 × 1 convolution, followed by a 3 × 3 convolution with the same feature size. The final ResNet convolution increased the feature size again to the initial size with a 1 × 1 convolution. For downstream convolutions, LeakyReLU with a fixed slope of 0.2 was used, and for upstream convolutions, ReLU was used. After each convolution, a group normalisation was performed, followed by the activation function. Additional weight standardisation was included because group normalisation together with weight standardisation performs better than batch normalisation for small batch sizes [22], [23]. The final activation function was a sigmoid function normalising the intensities between 0 and 1.

As training data was sparse, different augmentation techniques were considered to reduce the risk of overfitting and to ensure that the model was trained on different samples in each iteration. Therefore, during training, the data was randomly padded to fit the shape of 128 × 384 × 384 voxels and data augmentation was applied using affine transformations with random rotations in the range of [−45°, 45°], translations in the range of 39 voxels in the transversal plane, and scaling with a factor between 0.9 and 1.1. Images were normalised to [0, 1] in the range of [0, 1000] and [−1000, 4000] for MR and CT, respectively. The final transformed image was randomly cropped in the required shape specified in the next paragraph. For parameter optimisation, Adam, including decoupled weight decay, was applied with a maximum learning of 1 × 10^−3^, a weight decay factor of 1 × 10^−4^, and beta parameters of 0.5 and 0.999 [24]. The one cycle scheduler increased and decreased the learning rate over the number of iterations [25].

The hyperparameter search included two different loss functions, the group size of the normalisation, and the depth of the network. For the loss function, the $L_{1}$ loss was compared to a feature loss using a pretrained 3D model (ResNet3D) from the torchvision library [26], [27]. A fixed group size of 32 was compared to a fixed feature number per group of 32. Finally, the depth of the model was dependent on the image size. The input size of 128 × 128 × 128 was down-sampled seven times, resulting in a 1 × 1 ×1 bottleneck vector size. The second input size, 128 × 192 × 192, was down-sampled six times resulting in a bottleneck feature map size of 1 × 3 ×3. A Hyperparameter search was performed for all MRI sequences during the training phase. The maximum number of epochs was set to 200 to reduce the computation load.

**Figure 1:**
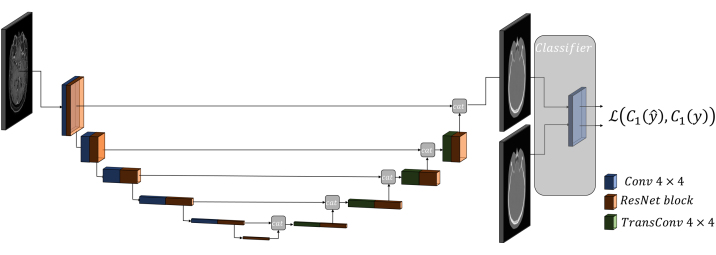
Final architecture schematics with the generated features of the pre-trained classifier. Blue blocks give the output of convolution layers and green blocks give the output of the transpose convolution layers. Orange blocks are the ResNet blocks.

The final model was trained with each MRI sequence separately (*i.e.* T1, T2, T1CM) as well as using all three MRI sequences (*i.e.* all). The network architecture can be seen in Figure 1. The performance of these four different trained models was again investigated on all three MRI sequences (*i.e.* validation dataset). Note that the images were not included in separate channels but to increase the sample size of the data. The iteration number was equally set for all training cycles resulting in deviating epoch numbers, as the data size was different for each sequence. The 44.5k iterations resulted in 500 epochs for the training using all sequences, 1850 epochs with T1 images, and 1350 epochs for T2 and T1CM. As the iteration number per dataset was smaller for the model trained with all sequences (between 12k and 16.5k for each sequence), the model was further trained with 1000 epochs (iteration for each sequence between 24k and 32k). All models were trained on a desktop PC with a GPU *NVIDIA Titan Xp* with 12 GB device memory.

### 2.4 Metric evaluation and statistics

For the sCT conversion, the SSIM and the MAE of thresholded regions were applied where the threshold was set to −200 HU and 300 HU of the pCT. The threshold value was also varied and plotted against the MAE. Additionally, the histogram of the HU intensities was used to group the voxels to equally sized bins of 100 HU and their MAE was calculated. A statistical evaluation was performed for the hyperparameter search and the influence of the sequences using the Friedman non-parametric test with the Nemenyi post hoc test. As significance level 0.05 was selected.

### 2.5 Treatment outcome analysis

The clinically approved rigid MRI to pCT transformations were applied neglecting an additional registration uncertainty. The clinical treatment plans were recalculated on the sCT in the RayStation plan evaluation module using the same stopping power-HU calibration curve. The existing spot patterns were recalculated on an sCT rigidly fused to the pCT.

The dose distributions computed for the pCT and the sCT were compared by dose volume histogram (DVH) parameters such as *D*_0.1cc_, *D*_1%_, *D*_2%_, *D*_50%_, *D*_mean_, *D*_D98%_, *D*_D99%_. Relative dose differences of plan's DVH values based on sCT (in percent) were compared to the respective CT plan values normalised to the prescribed dose per fraction (*D*_presc_), following Eq. (1):

(1) $$\Delta D(V)=\frac{D_{\mathrm{sCT}}(V)-D_{\mathrm{CT}}(V)}{D_{\mathrm{presc}}}\times 100$$

where *D*_CT_(*V*) and *D*_sCT_(*V*) are the respective dose volume parameters of volume *V*. The representation of the dose parameters is given in a violin plot, which – in addition to a boxplot – provides information on the distribution of the data. While the focus of the study was on the target structures, dose parameters of the OARs were also briefly reported.

Further, the spot difference between pCT and sCT was analysed by the distance in 3D space using a Python script in Raystation and is presented as a histogram.

## 3 Results

### 3.1 Hyperparameter search and metric evaluation

Table 2 shows the results of the hyperparameter search regarding the metric results. The SSIM was between 0.95 and 0.98 for all trained models. Using six down convolutions with a larger image crop setting performed better than the network with smaller image crops. The largest differences were observed for the MAE of the body because the network trained with fixed features per group and feature loss resulted in statistically better results compared to the others trained with $L_{1}$ loss or fixed groups. However, a significant difference was not present for the other metrics. Thus, the final model used for the sequence investigation was trained with fixed features per group, six down-convolution steps, an input image size of 128 × 192 × 192, and feature loss. The time to train the model was 8–10 h, depending on the parameter settings.

**Table 2:** Metric results of the hyperparameter search including loss, model depth/image size, and group size for the validation dataset. Bold values indicate best performance.

	MAE body [HU]		MAE bone [HU]		SSIM	
	mean ± std	[min, max]	mean ± std	[min, max]	mean ± std	[min, max]
Fixed features/group	**80.1 **±** 7.7**	[70.8, 95.2]	217.0 ± 25.9	[175.5, 261.7]	0.96 ± 0.01	[0.95, 0.97]
Fixed groups	86.4 ± 7.0	[77.5, 101.1]	**213.7 **±** 17.1**	[181.7, 246.4]	**0.97 **±** 0.01**	[0.95, 0.98]
Image size 128 × 128 × 128	85.6 ± 8.6	[74.3, 103.5]	234.3 ± 28.5	[191.5, 290.3]	0.97 ± 0.01	[0.95, 0.97]
$L_{1}$	82.3 ± 7.7	[73.1, 98.8]	221.7 ± 26.5	[181.6, 276.7]	0.97 ± 0.01	[0.96, 0.97]

Table 3 compares the model performance of models trained with only one MRI sequence to training based on all MRI sequences at the same time. The metric validation was similar for 98k iteration training for all sequences compared to the single sequence training. However, using models trained on one specific sequence (*i.e. T*_2_-weighted images) had significantly worse metric results (see Table 3). It took 20-24 h to train the final model.

**Table 3:** Detailed comparison of the validation dataset between models trained for each individual sequence and for all to the respective sequence for the final model configuration. Bold numbers indicate statistical test results below 0.05.

Iterations	Training set	Sequence	MAE body [HU]		MAE bone [HU]		SSIM	
			mean ± std	[min, max]	mean ± std	[min, max]	mean ± std	[min, max]
98k	All	T1	69.1 ± 5.7	[59.5, 78.9]	188.0 ± 15.7	[165.1, 211.8]	0.97 ± 0.00	[0.97, 0.98]
44.5k	All	T1	70.7 ± 5.2	[62.0, 79.7]	192.5 ± 15.5	[170.0, 216.8]	0.97 ± 0.00	[0.97, 0.97]
44.5k	T1CM	T1	**108.8 **±** 11.8**	[98.2, 133.4]	**345.4 **±** 41.4**	[299.4, 427.9]	**0.95 **±** 0.00**	[0.95, 0.95]
44.5k	T1	T1	68.1 ± 5.4	[59.6, 77.7]	186.9 ± 16.6	[161.1, 216.5]	0.97 ± 0.00	[0.96, 0.97]
44.5k	T2	T1	**108.7 **±** 5.4**	[104.2, 120.1]	**340.9 **±** 12.0**	[328.2, 355.2]	**0.96 **±** 0.00**	[0.95, 0.96]

98k	All	T2	67.3 ± 7.1	[58.0, 80.0]	176.2 ± 19.7	[153.0, 207.5]	0.98 ± 0.00	[0.98, 0.98]
44.5k	All	T2	70.1 ± 6.5	[61.8, 81.6]	182.9 ± 19.0	[160.7, 212.1]	0.98 ± 0.00	[0.97, 0.98]
44.5k	T1CM	T2	**115.9 **±** 14.4**	[97.5, 142.9]	**328.7 **±** 60.2**	[250.3, 429.0]	**0.95 **±** 0.00**	[0.94, 0.95]
44.5k	T1	T2	**134.6 **±** 15.1**	[114.6, 162.0]	**326.2 **±** 34.2**	[272.5, 366.9]	**0.96 **±** 0.00**	[0.95, 0.96]
44.5k	T2	T2	68.3 ± 7.3	[59.2, 80.5]	178.3 ± 23.0	[151.4, 209.5]	0.98 ± 0.00	[0.98, 0.98]

98k	All	T1CM	70.0 ± 8.4	[57.7, 80.0]	196.6 ± 23.2	[160.4, 226.7]	0.97 ± 0.01	[0.96, 0.98]
44.5k	All	T1CM	73.5 ± 8.7	[61.5, 83.9]	205.1 ± 23.0	[167.3, 230.5]	0.97 ± 0.01	[0.96, 0.98]
44.5k	T1CM	T1CM	71.6 ± 9.4	[57.8, 83.7]	205.7 ± 26.8	[157.2, 241.1]	0.96 ± 0.01	[0.95, 0.97]
44.5k	T1	T1CM	**172.9 **±** 24.1**	[139.5, 212.7]	**499.1 **±** 68.2**	[390.0, 605.3]	**0.93 **±** 0.01**	[0.92, 0.95]
44.5k	T2	T1CM	**147.6 **±** 22.3**	[116.5, 174.7]	**352.3 **±** 48.8**	[285.4, 423.3]	**0.94 **±** 0.01**	[0.93, 0.96]

**Figure 2:**
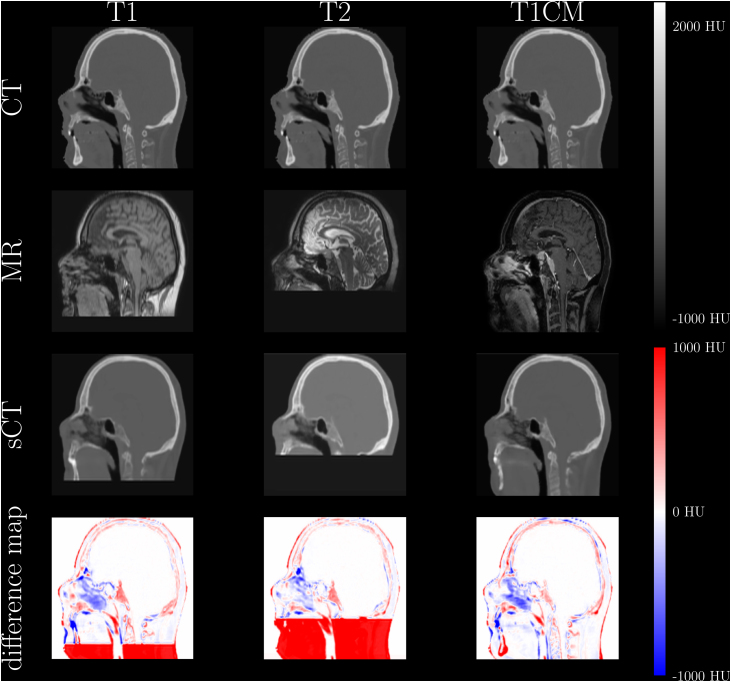
Generated sCTs with the corresponding MRI sequences (T1, T2, T1CM) and CT images for a representative patient in sagittal view. In the last row the difference maps between the sCTs and the pCT images are illustrated.

**Figure 3:**
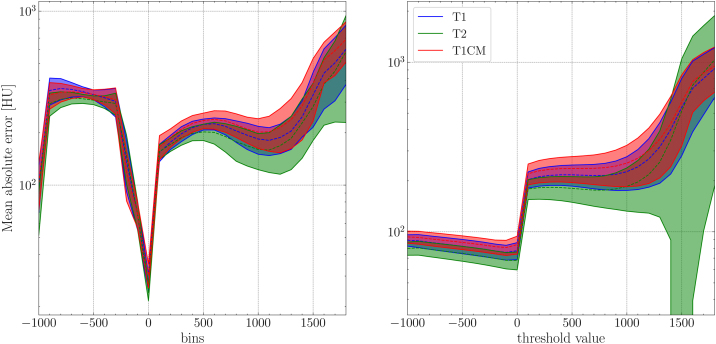
SCT MAE spectra including HU values in a bin size of 100 HU (left) or all voxels above a threshold value (right) which are both given by the *x*-axis. The shaded area indicates the region between two standard deviations.

The model applied to the test dataset was trained with all sequences and 98k iterations. The metric results of the test dataset can be found in Table 4. All metric results except for the SSIM were increased compared to the validation dataset results. Visual comparison for each MRI sequence of an example patient in sagittal view can be found in Figure 2 including an HU difference map between pCT and sCT. Despite the different contrast of each MRI sequence, the output sCT is of similar quality (see Table 4). Both MAE spectra in Figure 3 show a drastic increase of the MAE between 0 and 100 HU. Within the given bin size of 100 HU (*i.e.* bin spectrum), the largest MAE were present in the range of −900 to −300 HU, as well as for values larger than 1500 HU, in which range also in the threshold spectrum yielded the highest MAE.

**Table 4:** Metric results of the test dataset.

Sequence	MAE body [HU]		MAE bone [HU]		SSIM	
	mean ± std	[min, max]	mean ± std	[min, max]	mean ± std	[min, max]
T1	79.8 ± 5.9	[71.1, 86.5]	216.3 ± 29.6	[178.9, 278.5]	0.97 ± 0.01	[0.96, 0.97]
T2	71.1 ± 3.1	[66.7, 76.6]	186.1 ± 25.7	[155.4, 242.4]	0.98 ± 0.00	[0.97, 0.98]
T1CM	82.9 ± 6.1	[75.1, 93.4]	236.4 ± 41.4	[195.8, 331.4]	0.96 ± 0.01	[0.95, 0.97]

### 3.2 Treatment outcome analysis

**Figure 4:**
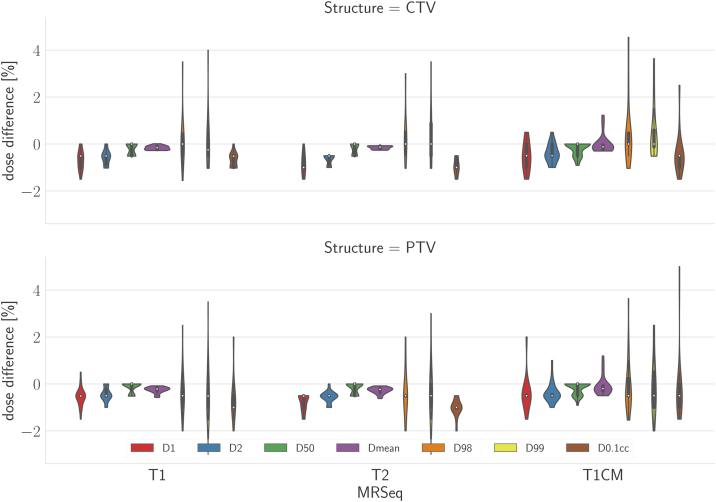
Difference of dose volume parameters for CTV (upper figure) and PTV (lower figure) comparing the sCTs (based on three different MRI sequences) with the pCT.

Figure 4 summarises the percentage dose difference between the recalculated (on sCT) and dose originally calculated on the pCT for the target structures clinical target volume (CTV) and planning target volume (PTV). The three different MRI sequences used as input for sCT generation are depicted separately. The varying input provided similar results regarding the investigated dose volume parameters, with a slight advantage for T1CM. On average, the percentage dose difference of all investigated dose parameters was below 1% comparing all sCT doses of the three input sequences with the respective dose values of the original plan.

All dose volume parameters for the brain were similar for sCTs retrieved from all investigated sequences, although T1CM had outliers compared to the pCT (over 2%) for *D*_1%_ and *D*_2%_ that were not observed for T2 and T1 based images. The difference in *D*_mean_ was below 1% for all sequences, while for *D*_0.1cc_ one outlier (4%) was present in all sequences, while all other dose parameter points were within ±2%. *D*_1%_ and *D*_2%_ for the brainstem and the optical nerves varied by ±3.5% for all input sequences, except for T1 where the values for optical nerves were higher (*i.e.* up to 5%) compared to the values of the treatment plan on the original CT.

**Figure 5:**
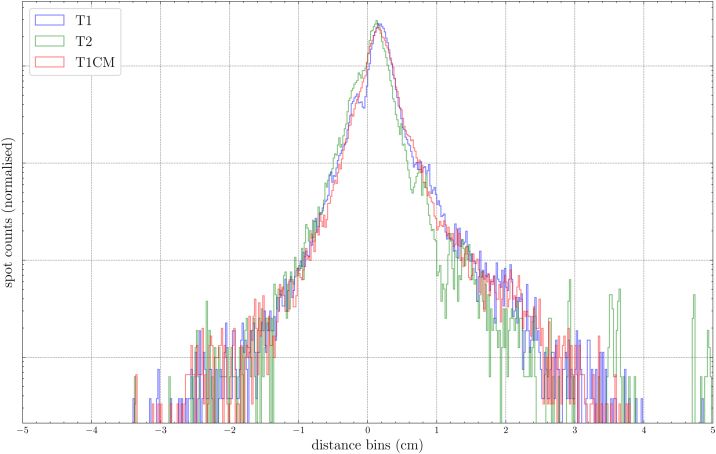
Spot difference between pCT and sCT spot positions for the different MRI sequence types. Values are normalised so that the area under the histogram integrates to unity.

The spot difference analysis shown in Figure 5 peaks at ±0.2 cm. For 95% of all spots, the absolute difference was below 0.6 cm and below 1 cm for 98% of all spots, for all input MRI sequences.

## 4 Discussion

In this study, a neural network was trained, capable of translating not only one but multiple MRI sequence types into sCTs for proton dose calculation. The general model design includes recent developments in the field of deep learning such as the Adam method, which uses decoupled weight decay [24], One Cycle learning rate scheduling [25], U-Net [19] combined with ResNet blocks [20], group normalisation with weight standardisation [22] and perceptual or feature-based loss functions [28]. The hyperparameter search included parameters like the model's depth, the layers of the pre-trained model used for the feature extraction and the correct group/feature numbers in the normalisation layers. The feature loss included only the first layer of the pre-trained model, which is mainly simple edge and corner features. Deeper representations were not investigated as this requires a high memory load, and the model was trained on RGB video data where a higher dimensional representation is questionable regarding medical data. This can be overcome by using a model pre-trained on medical data to make high-level features exploitable, but this was beyond the scope of this study.

The metric comparison showed no significant differences between the model trained with all MRI sequences and the model trained only for one particular MRI sequence. It has to be stressed that even the total number of iterations was higher for the final model (*i.e.* including all images), the number of iterations per sequence type was lower compared to the individually trained models. This shows that the model can be trained with multiple sequence types without affecting the performance of the single sequences. Theoretically, this could be extended to more than three sequences, requiring further investigations regarding the hyper-parameters. Further improvements could be achieved by using mixup regularisation to force the network to learn linear behaviours between samples [29], [30].

In comparison with literature, the metric results showed good agreement regarding mean values of the MAE for the body ranging from 30 HU to 100 HU [4], [6], [7], [12], [13]. It is worth noting that the MAE computation depends on different parameters, such as voxel resolution, calculation region, etc. Therefore, it is challenging to directly compare different models presented in the literature. For example, in our study, the MAE metric results of the different sequences favour the T2 sequences. This supports the findings of Qi et al. who compared multiple different sequences to determine the best suited sequence for sCT generation [7]. However, the focus of our study was not to determine the optimal sequence, which was hampered by a varying longitudinal field of view (FoV) between different sequences. For the processed images of this study, for example, the longitudinal FoV of T2 was shorter than for T1 and T1CM, which as a consequence, implied a reduced size of error-prone regions (*e.g.* spine or jaw). In this study, the main focus was to utilise all sequences acquired on the MR to build a robust and sequence independent model that performs on par with a model trained on a single sequence. Using the same number of iterations to train the models with all and only one sequence, showed that the performance is not weakened even if a sequence is less frequently applied during training (see Table 4). The increased number of iterations to 98k was performed to match the number of sequences applied during training, which resulted in a better performance for all individual sequences. The augmentation of training data with different sequences provides the possibility to apply the conversion independently of the sequence used during acquisition.

As outlined in Section 2, the total number of T2 images was smaller than the other sequences in the test dataset. In addition, some images were excluded as the T2 sequence did not cover the entire target structure due to the limited FoV of this particular sequence. Further, the performance of the test dataset was inferior to the validation dataset as many patients included had deformations and tumours in the nasal cavity region, which were not present in the training dataset.

Dosimetric comparisons of the recalculated treatment plans on the sCT with the original clinical plans showed average dose differences in the range of ±1% for the target structures and similar results for the brain. Larger differences were observed for smaller structures such as the brainstem or the optical system. However, the dose to such small regions is already affected by small deviations of the densities in the beam path due to their vicinity to the high dose regions covering the target. The steep dose gradients which, are necessary to spare these organs and which are the rationale for using proton therapy in the first place, are the reason for greater changes in the maximum dose parameters (*D*_0.1cc_ and *D*_1%_). The subsequent high impact on the dose volume parameters does not provide additional information on the sCT quality.

Shafai-Erfani et al. [11] investigated patients with base-of-skull tumours for an MRI-only workflow for proton therapy. In their study, the dosimetric evaluation (*i.e. D*_mean_, *D*_max_, *D*_10%_, *D*_50%_, and *D*_95%_) for the PTV was below 1% and up to 2% for the brain. When comparing our dosimetric results to literature, it has to be stressed, that most comparable studies [6], [11] created proton plans on a patient cohort that initially received photon therapy. The only exception being [13], where actual proton treatment plans were applied. Also in the presented study all involved patients received proton therapy, and the dosimetric comparison was based on clinically approved treatment plans. This affects also the performance of the model as the patients’ anatomy can vary more than in studies ignoring the more challenging conditions for actual proton therapy patients.

Within this study, the limitation of the model were also investigated. For a patient suffering from a sphenoid wing meningioma the dosimetric differences between pCT and sCT for CTV and PTV reached 20% for *D*_1%_ and *D*_0.1cc_, while *D*_mean_ and *D*_50%_ were below 1.5%. This was due to the model's performance in the nasal cavities region, which is inferior to other parts of the head.

Although the reported dose parameters are essential to judge the quality of the sCT, for particle therapy, the impact on the spot distribution is also crucial. In this study, spot position differences between pCT and sCT were calculated to compare the three input MRI sequences using Python scripts in RayStation. Only about 1% of all spots had a difference of more than 1 cm and about 5% had a difference higher than 0.6 cm, while the weight of the spots was not considered in Figure 5. In comparison, the proton beam spot size ranged from 6.8 mm for 252.7 MeV to 21 mm for 62.4 MeV.

Another issue, which accounts for sCT generation in proton therapy in general, is that immobilisation masks are hardly visible on MR images. Thus, the model is incapable of generating the masks’ material. However, in routine proton treatment, the patient position and the immobilisation devices used during CT and MR imaging are identical to the irradiation position, and the mask material is considered in the dose calculation process based on the pCT. In this study, in contrast to several others, the recalculated proton plans that are based on sCTs are compared with original pCTs, including the masks. This could be one reason, together with conversion uncertainties, why the spot difference histogram has two distinct peaks at ±0.2 cm and is not normally distributed.

## 5 Conclusion

The use of a universal sCT generator based on neural networks is the first step to enable translations of different MRI acquisition protocols. Thus, the quality of the produced sCT is independent of the input MRI sequence, which in turn does not hamper the clinical workflow. Dosimetric comparisons and spot difference maps showed a very good agreement between the treatment plans calculated on the initial pCT and the sCT. This study demonstrates, that the complexity of clinical applied proton plans from “real-world data” poses more challenges in an MRI-only workflow than currently displayed in the literature.

## Conflict of interest

The authors declare that they have no known competing financial interests or personal relationships that could have appeared to influence the work reported in this paper.
